# Deciphering Abbreviations in Malaysian Clinical Notes Using Machine Learning

**DOI:** 10.1055/a-2521-4372

**Published:** 2025-02-11

**Authors:** Ismat Mohd Sulaiman, Awang Bulgiba, Sameem Abdul Kareem, Abdul Aziz Latip

**Affiliations:** 1 Health Informatics Centre, Planning Division, Ministry of Health Malaysia, Putrajaya, Malaysia; 2 Academy of Sciences, Kuala Lumpur, Malaysia; 3 Faculty of Computer Science and Information Technology, University of Malaya, Kuala Lumpur, Wilayah Persekutuan, Malaysia; 4 MIMOS Berhad, Kuala Lumpur, Malaysia

**Keywords:** electronic health record, discharge summaries, word embedding, machine learning, natural language processing, health system management

## Abstract

**Objective** This is the first Malaysian machine learning model to detect and disambiguate abbreviations in clinical notes. The model has been designed to be incorporated into MyHarmony, a natural language processing system, that extracts clinical information for health care management. The model utilizes word embedding to ensure feasibility of use, not in real-time but for secondary analysis, within the constraints of low-resource settings.

**Methods** A Malaysian clinical embedding, based on Word2Vec model, was developed using 29,895 electronic discharge summaries. The embedding was compared against conventional rule-based and FastText embedding on two tasks: abbreviation detection and abbreviation disambiguation. Machine learning classifiers were applied to assess performance.

**Results** The Malaysian clinical word embedding contained 7 million word tokens, 24,352 unique vocabularies, and 100 dimensions. For abbreviation detection, the Decision Tree classifier augmented with the Malaysian clinical embedding showed the best performance (F-score of 0.9519). For abbreviation disambiguation, the classifier with the Malaysian clinical embedding had the best performance for most of the abbreviations (F-score of 0.9903).

**Conclusion** Despite having a smaller vocabulary and dimension, our local clinical word embedding performed better than the larger nonclinical FastText embedding. Word embedding with simple machine learning algorithms can decipher abbreviations well. It also requires lower computational resources and is suitable for implementation in low-resource settings such as Malaysia. The integration of this model into MyHarmony will improve recognition of clinical terms, thus improving the information generated for monitoring Malaysian health care services and policymaking.

## Introduction

MyHarmony is a natural language processing (NLP) system, which was built in 2016 to extract information from narrative text, such as diagnosis, procedures, and drugs. 1 2 3 It was developed by the Ministry of Health Malaysia to reuse clinical information for health service monitoring and planning. MyHarmony extracts information by recognizing clinical entities, codifies it to a clinical terminology standard like SNOMED CT, and stores it in a structured format for statistical analysis. The system applies a simple tokenization, n-gram, and a set of negations for codification.

One of the challenges of extracting information from electronic clinical notes is the abundant use of abbreviations, which can be ambiguous due to their multiple possible meanings. For example, MR can mean “mister” to address a person, “mitral regurgitation” of the heart valve, or “modified release” for drugs. Misinterpreted abbreviations have been shown to cause patient harm and mislead researchers and policymakers. 4 5 6 In Malaysia, 13 to 17% of physicians had experienced delays in giving medications, performing procedures, or making diagnoses due to abbreviation-related reasons, whereas 9 to 12% had made wrong diagnoses and given wrong therapies because of misinterpreted abbreviations. 5 In MyHarmony, extracting ambiguous abbreviations would result in inaccurate analysis.

NLP techniques to address abbreviations in clinical notes have been modernized with the use of word embeddings, 7 8 9 transformers, 10 and recently, Large Language Models (LLM). 11 12 New techniques allow context from surrounding words and therefore are reliant on the writing style and language of the corpus. This aspect is relevant to clinical notes that are known to differ from general domain texts or formal biomedical literature. 13 An example of a LLM specific to the medical domain is Med-PaLM 2 developed by Google, which scored 86.5% on a U.S. Medical Licensing Examination (USMLE) style question. 14

The evolution of these techniques has brought significant advancement but increasingly demanding computational requirements. The decision to use which technique relies on the resources available for implementation, either to deploy in a live environment during the clinical documentation process or as backend processing after storing clinical data. For example, LLMs such as GPT-3, BioGPT, and Med-PaLM 2, demand high-performance Graphics Processing Unit or even a Tensor Processing Unit to handle large numbers of parameters. Transformer-based models, like BERT 15 and its clinical derivatives including ClinicalBERT 16 and BioBERT, 17 offer more context awareness than word embedding but require specialized hardware and memory capacity. 18 19 20 Additionally, Med-PaLM 2 is currently restricted to specific countries, requiring approval for use via Google Cloud. 14

In contrast, word embeddings, such as Word2Vec, 21 GloVe, 22 and FastText, 23 are lightweight enough to run on a standard Central Processing Unit. In our use case, we chose to explore word embeddings to achieve our objective while meeting the constraints of our low-resource setting. Additionally, it is tailored to the Malaysian clinical context, and the informal writing style in clinical notes, written by non-native English-speaking clinicians in Malaysia.

The objective of our research was to develop and compare the performance of the Malaysian clinical embedding (MyCE) against the nonclinical and larger FastText embeddings (FTE) in the task to detect and disambiguate abbreviations in Malaysian electronic clinical notes. There were no previous studies that compared the performance of a machine learning model using word embedding from a general domain against clinical notes, except that of a formal biomedical literature. 24 The aim is to include abbreviation detection and disambiguation model in the MyHarmony NLP pipeline, thus improving its information extraction.

## Literature Review

Word embedding is a representation of a word and its relatedness to another word in the vector space. 8 Each word in the model is embedded with a context represented by vectors, where the vectors correspond to the relatedness of one word to another word in the model. This assumption is based on the distributional hypothesis, where words that are close together suggest some semantic relatedness. 25 Once trained, the word vector can be used as a feature for a machine learning model, such as in predicting a word as an abbreviation and what the abbreviation could mean.

The type of corpus plays a pivotal role in creating the word embedding model. This in turn influences the vocabulary size, word relatedness, and the dimension of the model. The bigger the corpus, the more likely it encompasses an extensive vocabulary and dimension. General word embeddings, like GloVe and FastText, are often trained with a corpus containing billions of words and more than 100 dimensions. However, researchers have shown that domain-specific word embedding, albeit smaller, would perform better than a general word embedding for domain-specific tasks. 24 For that reason, word embedding from the biomedical domain, specifically from formal biomedical literature, has been created and made publicly available. For example, BioWordVec was created using PubMed literature and Medical Subject Headings (MeSH). 26 Several state-of-the-art word embeddings using BERT have also been made available, such as BioBERT using PubMed abstracts and PMC full-text articles 17 and SciBERT using random samples from Semantic Scholar papers. 27 Specifically for clinical notes, ClinicalBERT was created using critical care notes from the Medical Information Mart for Intensive Care (MIMIC)-III database. 16

There are several challenges in choosing a suitable word embedding for a local implementation to address a specific task like addressing abbreviations. First, there are a limited number of word embeddings using clinical notes that are publicly available due to data privacy policies. 8 For example, BioWordVec was based on the word2vec model but trained on formal literature, which may not reflect the language and writing style in clinical notes. Additionally, publicly available word embeddings are usually trained from MIMIC-III critical care notes from Beth Israel Deaconess Medical Center in Boston, Massachusetts, United States, and may not reflect the context of Malaysian clinical notes.

## Methods

**Fig. 1:**
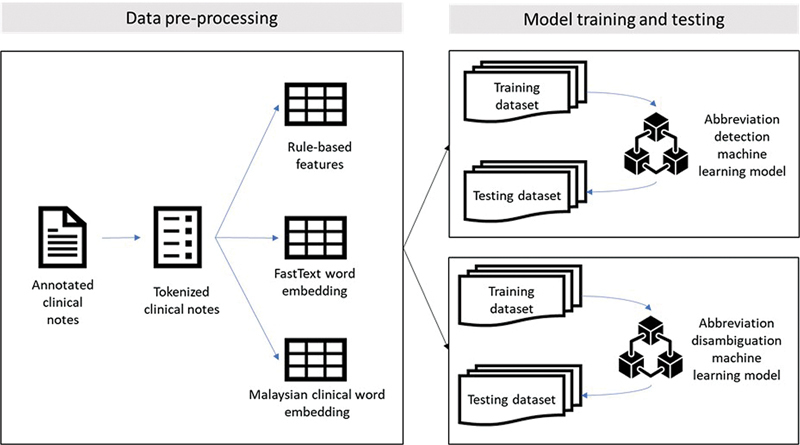
Design framework to detect and disambiguate abbreviations.

This research was conducted on an extract of Malaysian electronic discharge summaries from MyHarmony. It was approved by the relevant ethics committee. Fig. 1 illustrates the research framework for the detection and disambiguation of abbreviations.

### Data Preprocessing

A total of 29,895 anonymized clinical notes from January to December 2017 were extracted from MyHarmony. From the extracts, 1,102 were randomly sampled, of which 939 were Cardiology discharge summaries and 163 were General Medicine discharge summaries. Eleven medical graduates annotated the samples over 3 months using a web-based open-source annotation tool called Brat Rapid Annotation Tool (BRAT). 28 They were required to (1) annotate the abbreviations and (2) give the sense (or expansion) for each abbreviation found. Misspellings and punctuation errors were excluded from the scope of this study. The lists of abbreviations and their senses were standardized with UK English spelling for congruency.

**Fig. 2:**
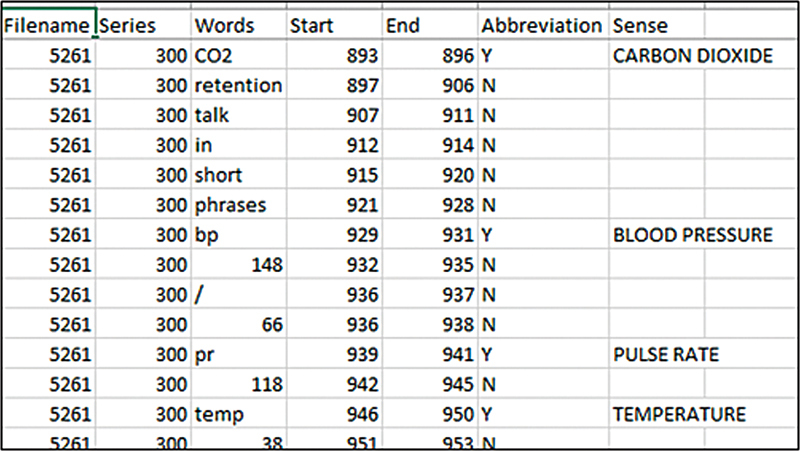
A screenshot of the tokenized dataset with labels for abbreviation and its sense.

The gold standard annotated clinical notes for abbreviations were tokenized. Each word was labelled either as an abbreviation or not. Those identified as abbreviations were assigned their respective senses. The tokenized clinical notes were assigned three types of features for comparative study: rule-based as a baseline, FTE, and the MyCE. Fig. 2 illustrates the tokenized clinical notes with labels for abbreviations and their senses.

### Features for Abbreviation Detection

There were 42 rule-based features for abbreviation detection based on the morphologies from the abbreviation, the word before, and the word after. The morphologic features are (1) the number of characters (integer); (2) the presence of special characters (Boolean): slash, negative, positive, alias; (3) the presence of period character at the beginning, in between, and at the end; (4) the presence of numbers at the beginning, in between, and at the end; and (5) the presence of character “X” at the beginning, in between, and at the end.

The FTE was trained on 16 billion tokens from Wikipedia and Web crawlers. It contains a 999,994 word vocabulary with 300 dimensions. For abbreviation detection, each token in the discharge summary was mapped to the vocabulary in FTE producing 300 features of word vectors. Out-of-vocabulary tokens, i.e., tokens without word vectors, were replaced with the constant 0 as recommended by Bojanowski et al. 23

The MyCE was created using the pretrained Word2Vec model 29 and trained on the entire set of discharge summaries extracted from MyHarmony. MyCE consists of seven million tokens, 24,352 word vocabulary and 100 dimensions. Similarly, out-of-vocabulary tokens were replaced with the constant 0.

### Features for Abbreviation Disambiguation

For abbreviation disambiguation, the rule-based features were similar to the study by Martínez and Jaber 9 and Wu et al. 7 A total of 42 features were used: the word itself, 10 from the word before and after, 10 from the word position, and 10 from the word direction, and 11 from abbreviation morphologies.

The dataset with FTE and Malaysian clinical word embedding has 11 features each, which represent the average vectors for the abbreviation and the 5-word window size. Null values due to out-of-vocabulary tokens were given a constant “0.” We averaged the vectors, which have been shown to represent the word vectors better, 7 9 as compared with using minimum and maximum values.

### Evaluation

We evaluated the embeddings on two classifiers, namely Decision Tree (DT) and linear Support Vector Machine (SVM). In previous studies, SVM consistently outperformed Naïve Bayes. 9 30 31 32 However, the comparison between SVM and DT was conflicting, which could be due to the different language used. 33 34

For abbreviation detection, the models were trained using the Cardiology dataset, internally validated using the 30% holdout method as the data were relatively large and would simulate the application on unseen Cardiology discharge summaries. The General Medicine dataset was used as an external validation.

For abbreviation disambiguation, five abbreviations were identified and listed in Table 1 . These five abbreviations have more than 200 instances. A similar research 7 discovered that the ideal minimal number of instances should be 200, and abbreviations with less than that would be too small and disproportionate to be trained and tested on. The three datasets were partitioned using 10-fold cross-validation.

**Table 1:** Five ambiguous abbreviations and distribution of their senses

Ambiguous abbreviation (instances)	Senses (instances)
CBG (202)	• Capillary blood glucose (197, 97.5%) • Capillary blood gas (5, 2.5%)
MG (1078)	• Milligram (1069, 99.2%) • Magnesium (9, 0.8%)
MR (247)	• Modified release (132, 53.4%) • Mitral regurgitation (84, 34%) • Mister (31, 12.6%)
PR (340)	• Pulse rate (314, 92.4%) • Per rectal (20, 5.9%) • Pulmonary regurgitation (6, 1.8%)
T (2537)	• Tablet (2,275, 89.7%) • Temperature (260, 10.2%) • Time (1, 0.04%) • To (1, 0.04%)

**Table 2:** Evaluation results for abbreviation detection

Classifier	Feature	Precision	Recall	F-score	AUC [95% CI]
Evaluation for cardiology dataset					
DT	RB	0.7605	0.6749	0.7151	0.9554 [0.954, 0.957]
	FTE	0.9177	0.9296	0.9236	0.9995 [0.958, 0.960]
	MyCE	0.9445	0.9594	0.9519	0.9996 [0.980, 0.982]
SVM	RB	0.6904	0.1885	0.2961	0.7633 [0.760, 0.767]
	FTE	0.9346	0.9041	0.9191	0.9923 [0.992, 0.993]
	MyCE	0.8837	0.8053	0.8427	0.9714 [0.970, 0.973]
Evaluation for general medicine (external) dataset					
DT	RB	0.8496	0.1050	0.1869	0.8428 [0.845, 0.855]
	FTE	0.7735	0.8511	0.8105	0.9220 [0.892, 0.901]
	MyCE	0.7928	0.9054	0.8454	0.9368 [0.912, 0.920]
SVM	RB	0.6875	0.0040	0.0079	0.5125 [0.655, 0.668]
	FTE	0.8243	0.7762	0.7995	0.9704 [0.928, 0.935]
	MyCE	0.7328	0.6660	0.6981	0.9284 [0.867, 0.877]

Abbreviations: AUC, area under the curve; CI, confidence interval; DT, Decision Tree; FTE, FastText word embedding; MyCE, Malaysian clinical word embedding; RB, rule-based features; SVM, support vector machine.

Due to the imbalanced classes for the senses, an Adaptive Synthetic (ADASYN) oversampling technique was applied during the preprocessing stage. 35 Applying an oversampling technique to address imbalanced classes in abbreviation disambiguation has not been seen in previous studies to the best of the authors' knowledge. The technique generates synthetic instances based on the density distribution of closest data points. ADASYN was applied on the datasets with the FTE and Malaysian clinical word embedding. The number of nearest neighbors is imputed as *k* = 5 and *β* = 0.5.

The performance of the different models was measured based on precision, recall, F-score (as the harmonic mean between precision and recall), area under the curve, and receiver operating characteristics (ROC) curve.

## Results

A total of 1,102 clinical notes were annotated and contained 243,679 word tokens, where 33,824 (19%) were abbreviations, and 7,640 (22.6%) of those abbreviations were ambiguous. The ambiguous abbreviations were constructed from 94 distinct abbreviations and 220 senses.

### Abbreviation Detection

The Cardiology dataset contained a total of 178,451 instances, where 70% (126,916) of the instances were used for model training, and 30% (53,535) were held out for internal validation. The classifier models were also externally validated on 65,228 instances from the General Medicine dataset.

**Fig. 3:**
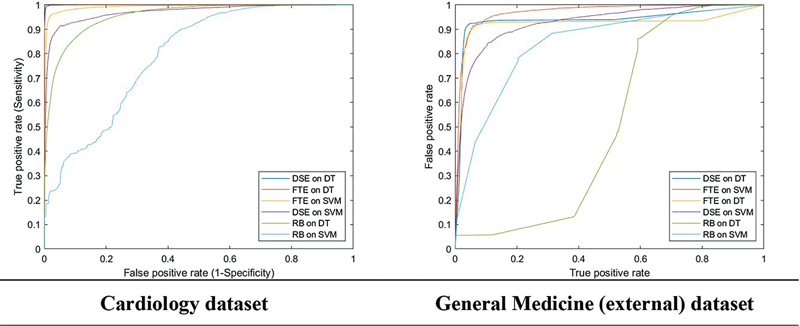
Receiver operating characteristic (ROC) curve for abbreviation detection. DSE, Malaysian clinical word embedding; DT, Decision Tree; FTE, FastText embedding; RB, rule-based; SVM, support vector machine.

Table 2 presents the comparison between the word embeddings with the DT and SVM classifier models and Fig. 3 shows the ROC chart. Overall, the DT model with the Malaysian clinical word embedding showed the best performance in detecting abbreviations (F-score of 0.9519). Word embeddings outperformed the rule-based feature in all aspects of the evaluation measures. When tested against the General Medicine dataset, there was a reduction in all aspects of the evaluation measure, with a reduction of F-score on the best-performing model from 0.9519 to 0.8454.

### Abbreviation Disambiguation

Table 3 shows the comparative results of the disambiguation models with different features. Models with the Malaysian clinical word embedding as features outperformed FTE most of the time, except for the abbreviation “PR.” For “PR,” the performance of a rule-based feature was at par with FTE and better than Malaysian clinical word embedding only by 1.26%. However, the F-score was 0.6518, indicating the need for better samples for the model.

**Table 3:** Evaluation results for disambiguating abbreviations

Feature	Precision	Recall	F-score	AUC [95% CI]
Abbreviation: CBG				
RB	0.8034	0.8007	0.8021	0.8561 [0.809, 0.903]
FTE	0.8102	0.8102	0.8102	0.8705 [0.825, 0.916]
MyCE	0.8804	0.9162	0.8980	0.9289 [0.893, 0.965]
Abbreviation: MG				
RB	0.9174	0.9450	0.9310	0.9624 [0.953, 0.972]
FTE	0.9243	0.9546	0.9392	0.9637 [0.954, 0.973]
MyCE	0.9814	0.9902	0.9858	0.9968 [0.994, 0.999]
Abbreviation: MR				
RB	0.8808	0.8636	0.8721	0.8615 [0.796, 0.921]
FTE	0.8831	0.8676	0.8753	0.8596 [0.792, 0.919]
MyCE	0.9885	0.9856	0.9870	0.9959 [0.990, 1.000]
Abbreviation: PR				
RB	0.6405	0.6635	0.6518	0.9980 [0.995, 1.000]
FTE	0.6405	0.6635	0.6518	0.9978 [0.995, 1.000]
MyCE	0.6241	0.6550	0.6392	0.9852 [0.973, 0.995]
Abbreviation: T				
RB	0.7839	0.7877	0.7858	0.8766 [0.865, 0.888]
FTE	0.7833	0.7866	0.7849	0.8764 [0.865, 0.888]
MyCE	0.9232	0.9342	0.9286	0.9734 [0.968, 0.978]

Abbreviations: AUC, area under the curve; CI, confidence interval; FTE, FastText embedding; MyCE, Malaysian clinical word embedding; RB, rule-based.

## Discussion

### Local and Domain-Specific Word Embedding

For both abbreviation detection and disambiguation from clinical notes, this research shows that word embedding as a feature performed better than the rule-based feature. Similar findings have been reported by Xu et al 36 when detecting abbreviations and Martínez and Jaber 9 when disambiguating abbreviations. Word embedding retains the semantic relatedness of the abbreviations and their surrounding words, whereas rule-based features do not. 8 Additionally, word embedding such as word2vec model is engineered using unsupervised learning technique saving time, as compared with rule-based features, which takes more time to be manually extracted.

Our study also suggests that domain-specific and locally built embeddings should be used rather than general embeddings, despite the smaller size. This suggestion concurs with Chen et al 24 and Charbonnier and Wartena 37 findings as domain-specific embeddings represent the relationship better than the corpus from the general domain when resolving domain-specific tasks. The results show that the Malaysian clinical word embedding was able to represent the meaning of the abbreviations better than FTE and thus enabled the classifier model to perform better.

### Implementation

**Fig. 4:**
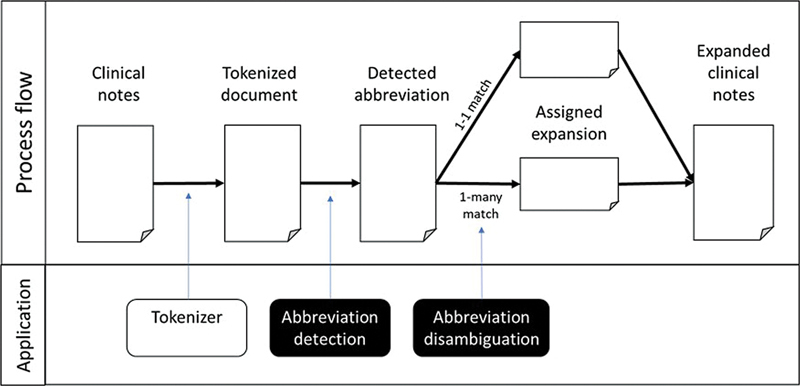
Framework to detect and disambiguate clinical abbreviations in the natural language processing pipeline.

The classification model is being incorporated into MyHarmony's NLP pipeline, as shown in Fig. 4 . After deciphering the abbreviations, an expanded clinical text is produced for the next task of recognizing and codifying clinical entities. This additional task in the NLP pipeline would reduce missed information extracted from the clinical text, thus improving the accuracy of information extraction and the statistical analysis it generates. 38

In managing the public health sector, monitoring and evaluating the health care services requires accurate data and information. Retrieving information from the first point of contact in patient care intends to reduce the workload of secondary data collection. Our approach may pave a new norm to collect data when answering ad hoc questions, rather than using the conventional method of questionnaires, quality registries, and data collection systems. A future study is being planned to investigate the cost of resources, time, data quality, and researchers' perception on our proposed approach compared with the traditional approach.

Furthermore, our model can also be applied in the local electronic health record (EHR). This is exemplified in the use case of the real-time clinical abbreviation recognition and disambiguation (rCARD). 33 rCARD was prototyped to detect abbreviations while clinicians make their entry and then suggest possible meanings for the ambiguous abbreviations. The rCARD model uses SVM classifier and was reported to take between 0.630 and 1.649 milliseconds for the clinicians to resolve the ambiguous abbreviations. This same approach is being proposed as the EHR is being implemented throughout public health hospitals and clinics in Malaysia. As clinicians document, abbreviations are automatically detected and highlighted. The clinicians may be given the choice of possible meanings for confirmation. Deciphering abbreviations at the point of entry would improve documentation quality, thus supporting safer continuity of care during follow-up visits. Unambiguous documentation simplifies and increases the accuracy of data retrieval for research, clinical program evaluation, and policymaking.

Additionally, the Malaysian clinical word embedding can be used as a feature to resolve other clinical NLP problems specific to the Malaysian context. A potential area of application is incorporating abbreviation detection and disambiguation models to automate text summarization. This is particularly useful in clinical settings when drafting discharge summaries and referral letters for patients. Another example of application is incorporating the model into speech recognition software that transforms speech-to-text to capture clinical entries. 39 The software can then decipher abbreviations into the correct expansion, thus improving the quality of documentation and reduce misinterpreted abbreviations.

### Limitations

This study is not without limitation, as the Malaysian clinical word embedding and the classification models involved only the Cardiology and General Medicine disciplines, which restricts the generalizability of the model if applied to other disciplines. This study showed a reduction of F-score from 0.9519 to 0.8454 when the abbreviation detected model was applied to the General Medicine clinical notes. This reduction is likely due to unseen abbreviations and context. The same outcome can be expected when applied to a surgical-based dataset or other disciplines. However, as more datasets from various disciplines are retrieved for training of the models, this limitation is expected to be overcome.

It is also unclear how the Malaysian clinical word embedding and the models would perform in clinical notes other than discharge summaries. The type of clinical notes may be a factor in the extent of abbreviations used. Discharge summaries are thought to be better structured and better written compared with inpatient notes and admission notes. 13 Applying the model to EHR systems that contain admission notes, inpatient notes, outpatient notes, and procedural reports may need further research and evaluation. One reassurance from this research is that the method established here can be replicated and extended with more robust word embedding and classification models by including other types of clinical notes.

## Conclusion

A domain-specific word embedding such as the Malaysian clinical word embedding was able to detect and disambiguate abbreviations in the Malaysian electronic clinical notes better than FTE and the rule-based model. The research also shows that using word embedding with traditional machine learning algorithms can decipher abbreviations well and therefore suitable for implementation in low-resource settings such as Malaysia. This is the first clinical word embedding for the Malaysian context and can be experimented on to resolve other NLP problems, such as machine translation, speech and text recognition, and text summarization. This research is also the first to develop machine learning models to detect and disambiguate abbreviations using Malaysian clinical notes. Future works involve developing a more robust Malaysian clinical word embedding by including discharge summaries from other clinical disciplines; implementing the model in EHRs to prevent misinterpretation of abbreviations during patient care and improving the data quality for health care management and financing; as well as comparing with large language transformer models and its feasibility in low-resource environments.
